# Dolphin Sounds-Inspired Covert Underwater Acoustic Communication and Micro-Modem

**DOI:** 10.3390/s17112447

**Published:** 2017-10-25

**Authors:** Gang Qiao, Yunjiang Zhao, Songzuo Liu, Muhammad Bilal

**Affiliations:** 1 Acoustic Science and Technology Laboratory, Harbin Engineering University, Harbin 150001, China; qiaogang@hrbeu.edu.cn (G.Q.); zhaoyunjiang@hrbeu.edu.cn (Y.Z.); bilal@hrbeu.edu.cn (M.B.); 2 College of Underwater Acoustic Engineering, Harbin Engineering University, Harbin 150001, China

**Keywords:** underwater acoustic communication, bionic, underwater acoustic modem, micro-modem, covert, dolphin sounds

## Abstract

A novel portable underwater acoustic modem is proposed in this paper for covert communication between divers or underwater unmanned vehicles (UUVs) and divers at a short distance. For the first time, real dolphin calls are used in the modem to realize biologically inspired Covert Underwater Acoustic Communication (CUAC). A variety of dolphin whistles and clicks stored in an SD card inside the modem helps to realize different biomimetic CUAC algorithms based on the specified covert scenario. In this paper, the information is conveyed during the time interval between dolphin clicks. TMS320C6748 and TLV320AIC3106 are the core processors used in our unique modem for fast digital processing and interconnection with other terminals or sensors. Simulation results show that the bit error rate (BER) of the CUAC algorithm is less than 10${}^{-5}$ when the signal to noise ratio is over ‒5 dB. The modem was tested in an underwater pool, and a data rate of 27.1 bits per second at a distance of 10 m was achieved.

## 1. Introduction

With the growing demand for marine development, underwater acoustic communication (UAC) and networks have been used in many fields, such as marine environmental monitoring, natural disaster warning, etc [1,2]. Furthermore, military applications include communication between divers, underwater unmanned vehicles (UUVs), submarines and wireless sensor networks, where the main focus is to lower the probability of detection and interception [3,4].

Based on the different modulation methods, the modems are divided into several categories including quadrature phase shift keying (QPSK) modems, frequency shift keying (FSK) modems, quadrature amplitude modulation (QAM) modems, and so on. A compact and low power micro-modem [5] designed by WHOI has the capability to perform low-rate frequency-hopping frequency-shift keying (FH-FSK), variable rate phase-coherent keying (PSK) and long base line navigation. As a successor, the WHOI micro-modem-2 [6] is significantly more capable, allowing new, advanced capabilities such as enhanced communication rates and underwater network deployments. Yan, H. demonstrated the real-time capabilities with both a floating point and a fixed-point DSP with QPSK modulation and described its detailed implementation in [7]. Aydinlik, M. presented the implementation of the physical layer of a reconfigurable underwater acoustic modem using QPSK modulation [8]. Blueprint Subsea developed the SeaTrac modem with QPSK modulation based on front-end specifications by Newcastle University in [9]. Previously, we focused on remote and robust underwater acoustic communication modems; we have developed and tested our modems, such as HEU OFDM-modem with QPSK modulation for UAC and networking [10,11,12].

Compared to QPSK modulation, FSK modulation is more widely used in modems. A low-power acoustic modem for dense underwater sensor networks using FSK modulation was developed by the University of Southern California in 2006 [13] and was tested by transmitting and receiving bits in the air. Vasilescu, I. designed a modem named Aqua Node mainly used in underwater wireless sensor networks and verified its performance in a lake, river, and ocean [14]. Parsons, G.S. presented an ultrasonic communication system for biotelemetry in extremely shallow waters by using the FSK modulation scheme in [15]. Cario, G. et al. developed a low-cost underwater acoustic modem called SeaModem with MFSK modulation which is reprogrammable in both the physical and network-layer [16]. Li, B. et al. developed a QAM-based MIMO Orthogonal Frequency-Division Multiplexing (OFDM) underwater acoustic modem in [17].

Communication between divers and their commander needs to be kept highly secure and the location of activity has to be kept secret. Using simple UAC, transmitted messages are more vulnerable to being intercepted by the enemy and the location can be easily traced. This calls for covert underwater acoustic communication which keeps the communication between divers highly secure and undetected by eavesdroppers. Some papers [18,19,20] have proposed using or simulating marine mammal sounds as the communication carrier to realize communication.

The primary purpose of this article is to design a Portable Biomimetics-based Covert (PBC) UAC micro-modem and realize covert communication by using real dolphin sounds. The communication carrier waveform in the PBC micro-modem is natural dolphin or whale sounds. In the specific communication process, the dolphin signal will be selected according to the actual situation to realize the effect of covert communication. For example, local dolphin calls should be selected for communication in different sea areas, and the seasonal dolphin calls should be used for communication in different seasons. In order to improve the communication speed and restrain the multipath interference between adjacent symbols, low correlation dolphin calls should be selected. The dolphin- and whale-call samples used in the communication algorithm are stored in the SD card in advance. In the PBC micro-modem, we implemented a variety of bionic communication algorithms as described in [18,21,22] according to different types of dolphin and whale sounds. It brings a higher degree of algorithmic freedom for practical applications. The operator can change the calls library and communication algorithm which can be based on different regions and implementations for different tasks.

The remainder of this paper is organized as follows: A comparison of developed devices, commercial devices and the PBC modem is described in Section 2. Section 3 discusses the design of the PBC micro-modem. The bionic covert communication algorithm and its implementation are described in Section 4. The pool experiment and results are analyzed in Section 5. Finally, we summarize our design and provide directions for future work in Section 6.

## 2. Related Work

With the evolution of technology and the demand for diversification, more and more requirements have emerged in Underwater Wireless Sensor Networks (UWSN). Nam, H. described an energy-aware acoustic modem and tested its performance in underwater experiments in [23]. An omni-directional underwater acoustic micro-modem based on a low-power microcontroller unit has been described in [24]. In 2012, Antonio, S. et al. made a breakthrough in low power consumption by inventing an ultra-low power and flexible acoustic modem which requires only 10 μW in standby mode and consumes extremely low power during reception and transmission [25]. Due to the high cost of modems, some researchers have focused on reducing their cost. Sanchez, A. et al. [26] started their study with the most critical component from a cost perspective—the transducer followed by other similar works in [27,28].

A few companies are actively researching UAC modems and have developed many commercial modems. The Atm series modem [29] made by Teledyne Benthos can support a maximum data rate of up to 15.36 kbps and the maximum working distance can reach 6 km. LinkQuest UWM series modem [30] can support a maximum data rate of up to 17.8 kpbs and the maximum working distance can reach 10 km. EvoLogics has developed a series of modems which employs Sweep-Spread Carrier (S2C) technology to realize communication [31].

Based on the comparative analysis of an underwater acoustic modem collated by Sendra, S. and Lloret, J. in [32,33], we have selected some representative devices and made a comparison table as shown in Table 1. These comparisons are performed in terms of modulation, areas of application and characteristics.

As we can see from Table 1, the main developing trends of the UAC modem applied to UWSN are low power consumption, low cost, miniaturization and so on. However, basically, no modem is concerned about the Covert Underwater Acoustic Communication (CUAC) between them. Different from the modems presented above, our PBC modem adopts unconventional modulation mode.

Additionally, the PBC modem focuses on the application of CUAC among UWSN nodes, divers or UUVs by using real dolphin or whale sounds and mimicking the sounds of sea creatures which is different from existing modems.

The dolphin and whale call samples used in the communication algorithm are stored in the SD card in advance which makes the selection of communication signals more flexible. For example, in the specific communication process, sound signals will be selected according to the actual situation in order to realize covert communication. Local dolphin calls should be selected for communication in different sea areas, and seasonal dolphin calls should be used for communication in different seasons.

These unique features make our PBC modem significantly different from existing developed and commercial modems.

## 3. Modem Design

**Figure 1:**
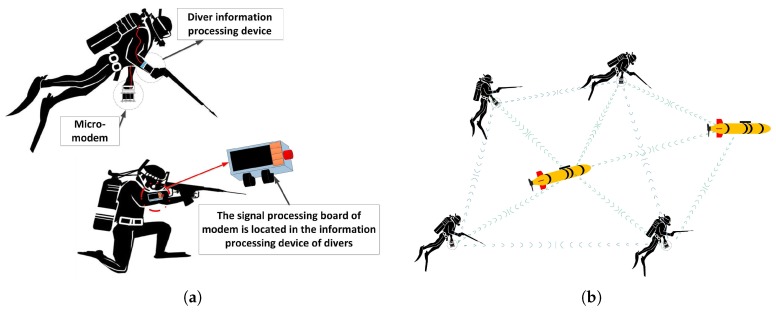
(**a**) Frogmen equipped with modems; (**b**) Communication among divers and UUVs over micro-modems.

Our modem is designed specifically for divers to communicate with their partners or UUVs securely as shown in Figure 1. On one hand, the communication algorithm needs to feature low probability of detection and interception. On the other hand, the modem hardware should be portable and consume low power. In this section, we describe the modem hardware design. The hardware of the modem is composed of two parts including electronic and transducer elements connected by a sealed tube. The transducer is used to transmit and receive communication signals. The electronic element has a signal processing board and power amplifier covered by a waterproof housing. Since the maximum working depth for the diver is less than 100 m, we use PVC-based housing, which is cheap and lightweight.

### 3.1. Modem Structure Design

**Figure 2:**
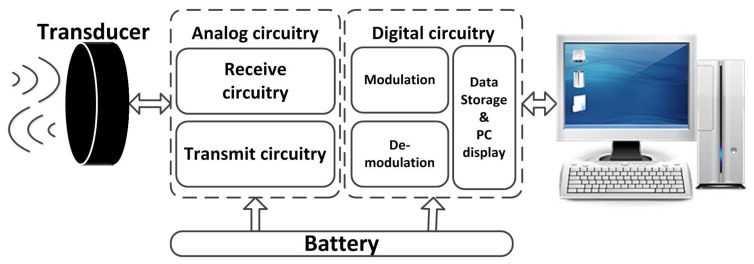
Traditional modem block diagram.

The conventional modem structure block diagram is illustrated in Figure 2. It consists of analog and digital circuit boards which can be connected with external devices such as a personal computer (PC) by the universal asynchronous receiver and transmitter (UART).

**Figure 3:**
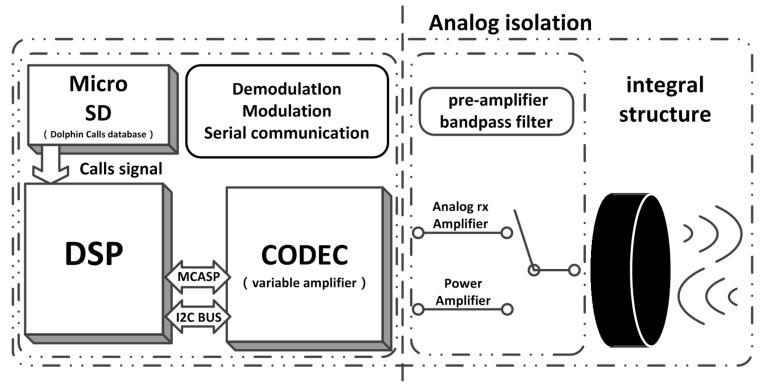
PBC micro-modem block diagram.

In our design, the analog and digital circuits are put together to reduce the volume of the hardware. It transmits the instructions via UART and acquires the audio signal by using the CODEC and Enhanced Direct Memory Access (EDMA) modules. We use Inter-Integrated Circuit (IIC) bus to control the TLV320AIC3106 [34]. The design of the portable bionic covert UAC micro-modem is finalized based on the above mentioned hardware and software and by realizing the bionic algorithm. Figure 3 shows the overall PBC micro-modem block diagram architecture.

### 3.2. Digital Domain Processing

#### 3.2.1. Core Digital Signal Processor

The core signal processor in our design is TMS320C6748 [35]. It is a low-power application processor based on a C674x DSP core. This DSP consumes significantly lower power than other members of the TMS320C6000TM platform of DSPs; furthermore, the rich user interfaces and high processor performance make it a better choice. Taking into account the requirements of the design and performance of the chip, the TMS320C6748 has many characteristics. Under the highest core frequency, the computing ability of the DSP chip can be up to 3648/2746MIPS/MFLOPS, which is quite helpful for the modulation and demodulation of the signal. The kernel voltage is as low as 1.0–1.2 V, and the I/O interface voltage is 1.8 V or 3.3 V. According to the manual, the total power consumption is less than 450 mW [35]. Through the system Power Sleep Control (PSC), in idle mode, the system power consumption can be as low as 10 mW, which makes this chip very suitable as the core of the underwater communication node. Low-power design can make the size of the modem battery cabin smaller and thus easier to carry. The number of independent DMA channels is 64. It can operate at EDMA3, which provides a precondition for the high-speed transmission of data. For the real-time communication system, data transmission based on EDMA is the premise of the whole system.

#### 3.2.2. Analog and Digital Signal Convertor

The analog to digital converter (ADC) and digital to analog convertor (DAC) for the modem is TLV320AIC3106. It is a low-power stereo audio codec with a stereo headphone amplifier, as well as multiple inputs and outputs programmable in single-ended or fully differential configurations. The sampling rate of the stereo audio ADC and DAC can vary between 8 kHz and 96 kHz. Extensive register-based power control is included, enabling stereo 48 kHz DAC playback as low as 15 mW from a 3.3 V analog supply, making it ideal for the portable battery powered UAC modem. The TLV320AIC3106 contains integrated microphone bias, a digitally controlled stereo microphone preamplifier, and automatic gain control (AGC), with mix/mux capability among the multiple analog inputs making the input SNR and the output SNR reach 102 dB and 92 dB, respectively.

In this design, AIC3106 communicates with DSP through the McASP and IIC interface. DSP sends a control signal to AIC3106 through the IIC interface. The data received or sent by AIC3106 is transmitted through the McASP serial interface to DSP.

### 3.3. Analog Domain Processing at Transceiver

**Figure 4:**
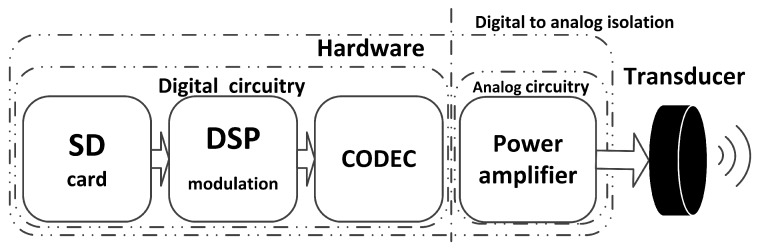
Functional diagram of the transmitter.

The analog circuit receives the signal from the digital circuit at which point the digital signal is changed into analog signal through AIC3106. After being amplified by the power amplifier, the signal is transmitted by the transducer as shown in the following Figure 4.

**Figure 5:**
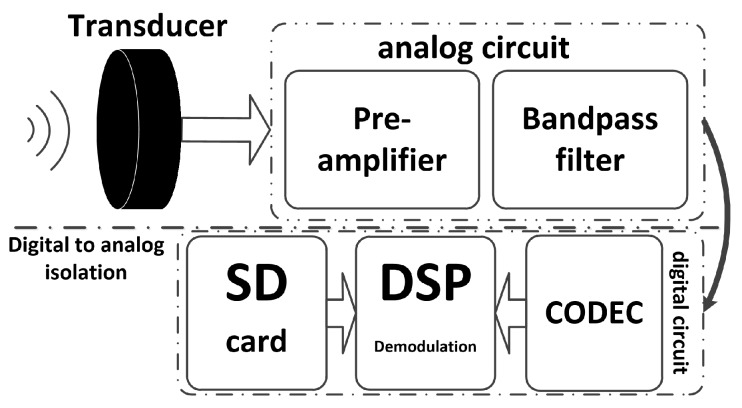
Functional diagram of the receiver.

At the reception, first the acoustic signal is transformed into a weak electronic signal by the transducer, then the preamplifier of the analog circuit will amplify the signal. Finally, the amplified results are transmitted to the digital circuit where they wait for CODEC perform the next operation. The process is shown in Figure 5.

#### 3.3.1. Power Amplifier

Piezoelectric transducer excitation has been previously carried out mainly using three power amplifier types: Class-B; class-D; and class-AB. When the class-B power amplifier becomes functional, positive and negative channel transistors usually remain in a closed state unless there is a signal input, which can be elaborated as when the positive phase signal is received, only the positive phase channel starts working while the negative phase channel remains closed. Furthermore, the two channels will not work simultaneously to avoid any power loss in the part where there is no signal. The use of a commercial class-AB amplifier can avoid the drawbacks of the class-B amplifier. Exploring the actual need and selecting the best available power amplifier, we decided to use the class-AB power amplifier in this design. The TDA2030A is a monolithic IC, intended for use as a low-frequency class-AB amplifier. With VS max = 44 V, it is particularly suitable for more reliable applications without regulated supply and for 35 W driver circuits using low-cost complementary pairs. The TDA2030A provides high output current and has very low harmonic and cross-over distortion. Furthermore, the device incorporates a short circuit protection system comprising an arrangement for automatically limiting the dissipated power so as to keep the working point of the output transistors within their safe operating area. A conventional thermal shut-down system is also included.

Normally, a class-D power amplifier is more efficient than a class-AB power amplifier. However, the class-AB amplifier peripheral circuit is relatively simple, which is more conducive to the miniaturization of the modem. In addition, the output audio quality of the class-AB power amplifier is better and less noisy than that of the class-D amplifier. With the requirement of modem miniaturization, it is difficult for class-D power amplifiers to achieve both low interference and circuit scale miniaturization at the same time and it may produce high-frequency harmonics. In general, the duration of underwater diving activity is several hours. Even though class-AB power amplifiers are less efficient than class-D power amplifiers, they can still meet this demand.

#### 3.3.2. Analog Amplifier of Reception

**Figure 6:**
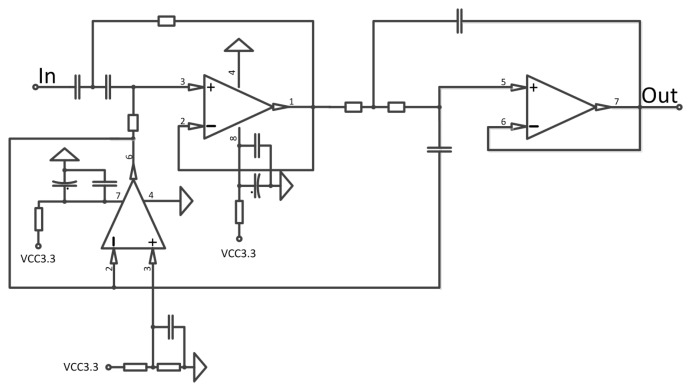
Analog processing stage schematic.

The transducer converts an acoustic wave signal to a differential voltage signal; however, the voltage signal is quite weak. For long distance transmissions, it is only a few tens of micro volts. However, in some cases, voltage can reach tens of millivolts, which exceeds the preamplifier requirements. At the same time, the programmable gain is very important for the realization of the actual project. Figure 6 shows the analog amplifier of the reception schematic.

The OPA348 series amplifiers are single supply, low-power, CMOS op-amps in micro packaging. Featuring an extended bandwidth of 1 MHz, and a supply current of 45 μA, the OPA348 series is useful for low-power applications on a single supply of 2.1 V to 5.5 V.

A low supply current of 45 μA and an input bias current of 0.5 pA make the OPA348 series a suitable candidate for our modem.

Instead of a simple pre-amplifier, the analog reception amplifier in this modem includes band pass filtering and an adjustable gain amplification. As the application’s environment demands a wideband filter, we use an analog amplifier to build a band pass filter by using a Butterworth low-pass filter and a second-order Butterworth high-pass filter, whose range is adjustable. The reason for choosing it is the flexibility to control the cut off frequency.

The amplifier programmable control amplification is less than 40 dB if the frequency of the received signal is above 1 kHz. The signal could be amplified the second time in the AIC3106.

### 3.4. Implemented Micro-Modem

**Figure 7:**
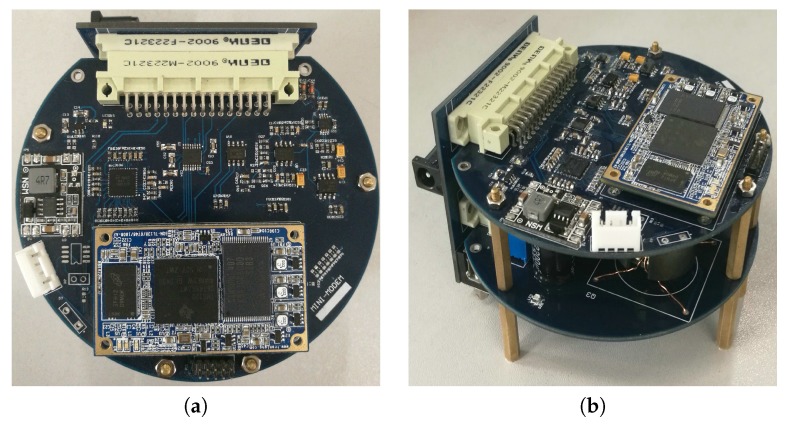
Experimental prototype of the micro-modem; (**a**) Digital board of the micro-modem; (**b**) Modem hardware.

The hardware design of the underwater acoustic modem is shown in Figure 7, which is cylindrical in shape with a diameter of 10 cm and a height of 6.2 cm.

## 4. Bionic CUAC Scheme

The main purpose of this design is to communicate covertly between divers or divers and UUVs. When the divers are closer to the target and the instructions are still on the way, the divers are more vulnerable to being detected if the target has diver detection sonar. The application of bionic communication will make the enemy misjudge the diver’s action as the communication signal will be excluded as it sounds like cetacean calls.

We modulate the signal based on the time interval to overcome the sound exposure derived from traditional fixed carrier modulation, treating the communication signal as ocean biological noise to achieve covert communication.

In this section, the main content is divided into two parts, the first part is the algorithm simulation and realization while the second part is the software design and algorithm implementation process.

### 4.1. Algorithm Simulation and Realization

This section introduces the specific process of algorithm implementation while referring to the literature [18] which is about covert UAC using dolphin sounds.

**Figure 8:**
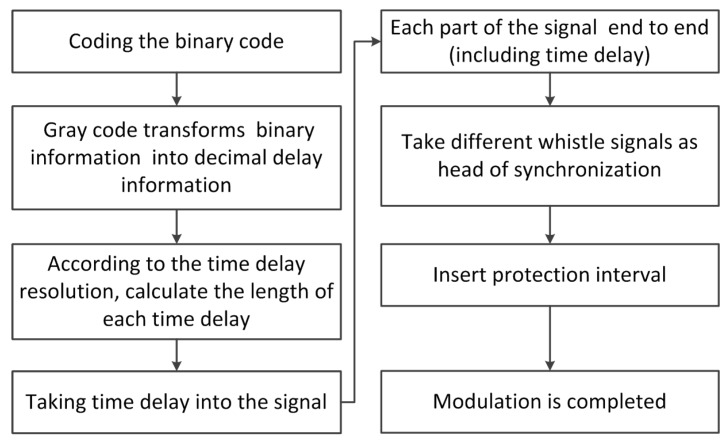
Process flow chart of bio-signal modulation.

**Figure 9:**
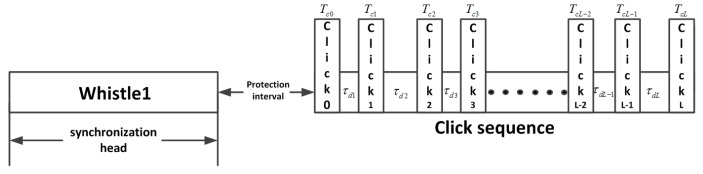
Frame structure of the mimic bio-signal combined with whistle and clicks.

In the modulation process, the data from the serial port is encoded first and each six bits are converted to decimal digits. The length of the time delay is calculated according to the time delay resolution. Delay difference is modulated between the interval of two signals according to the calculation. After assembling all click signals and time delay together, the synchronization head and the protection interval are added to the header of the signal. The process flow chart of bio-signal modulation is shown in Figure 8. The frame structure of the complete mimic bio-signal combined with whistle and clicks is shown in Figure 9.

$\tau_{di}$(*i* = 1,2,3,⋯,L) is the is the coding time, which refers to the time delay difference between two click signals. Each click signal time duration is represented by $T_{cj}$(*j* = 0,1,2,⋯,L). For each *n* bits of encoded information, the encoding time is divided into ($2^{n}-1$) parts, and the time duration of each part is Δτ that we set to 1 ms. Therefore, the time delay can be calculated as:

(1) $$\tau_{d}=\Delta \tau \cdot k\;,\;k=0,1,\cdots ,2^{n}-1.$$

Here, *k* is the decimal information transformed by Gray code from a binary information source. For example, if each encoded element has seven bits of information, the encoding time would be divided into 127 parts. If the source information is 1010111, after Gray encoding there would be *k* = 65. Therefore, the time delay $\tau_{d}$ would be 65 ms.

As the width and encoding time of each click is variable, the communication data rate of the system can be calculated as:

(2) $$\;v=L\times n/\sum_{l=1}^{L}\left(T_{cl}+\tau_{dl}\right)$$

At the receiver, side synchronization is achieved by the whistle. After synchronization, the protection interval and the first click signal are removed in order to reduce the time of operation as it does not carry any information and the length is known ($l_{0}$).

The length of the signal in the first correlation operation is $l_{1}+l_{t\max}$. The starting position is identified by correlation peaks of the last iteration. The signal length in the *i*-th correlation operation is $l_{i}+l_{t\max}$. In this way, if there are L + 1 clicks in a complete signal, the system needs to perform correlation L times. Therefore, there is no need to perform correlation for the first click signal and ensure that the length of the signal results in the shortest first operation signal with no loss of information.

**Figure 10:**
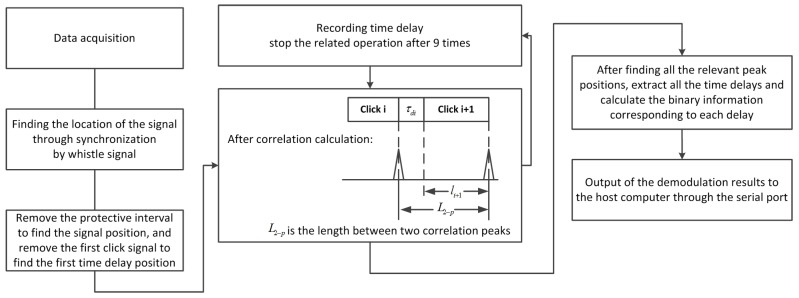
Process flow chart of bio-signal demodulation.

The correlation peak position minus the length $l_{i}$ is the corresponding time delay. We can obtain the transmitted data after converting the decimal data into binary data. Finally, the system will upload data to the host computer to be displayed through the serial port. The whole process flow chart is shown in Figure 10.

**Figure 11:**
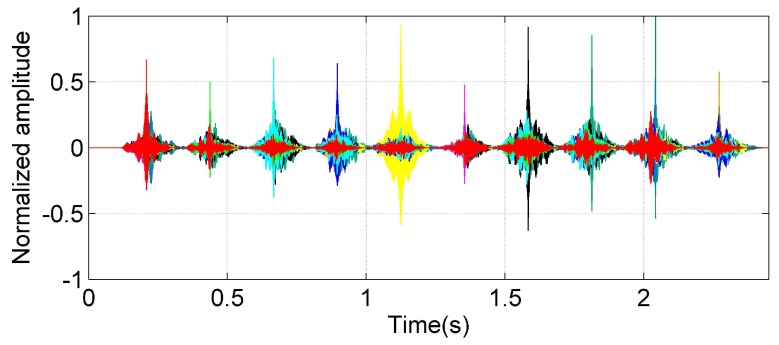
Correlation output of clicks.

The click signals we selected have excellent autocorrelation as shown in Figure 11. Different colors represent the correlation output between different click signals and the click signals group. Each peak reflects the autocorrelation of the click signals. Table 1 lists the normalized cross correlation and autocorrelation coefficients of each signal.

From Table 2, we can ascertain that the correlation coefficients between the signals are less than 0.43.

**Figure 12:**
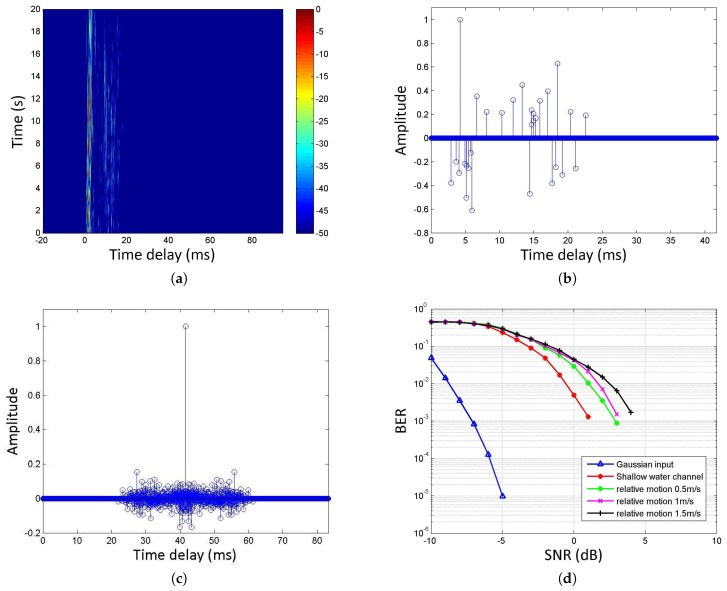
Algorithm simulation conditions and results; (**a**) Time-varying channel impulse response; (**b**) Channel impulse response estimated by the MP method using a dolphin whistle; (**c**) Virtual channel after virtual time reversal mirror (VTRM) by using the estimated channel impulse response; (**d**) bit error rate (BER) of demodulation results.

We obtained the bit error rate (BER) curve of the algorithm under white Gaussian noise by simulation. At the same time, we used the real lake channel to test the performance of the algorithm, and used the matching pursuit (MP) [36] algorithm to estimate the channel, and then compensate the signal through the virtual time reversal mirror (VTRM) [37]. Moreover, in order to verify the performance of the proposed algorithm under the influence of Doppler, we simulated the invariant Doppler factor and various relative motion velocities by resampling, and obtained the bit error rate curve. The results are as shown in Figure 12.

The underwater channel is derived from Songhua Lake in China. The water depth was about 40 m. The transmitter was about 13 m and the receiving hydrophone was about 12 m below the boats with a distance of 820 m. The simulation results show that the BER is less than 10${}^{-5}$ when the signal-to-noise ratio is greater than ‒5 dB with the white Gaussian noise. Furthermore, it can be seen from the simulation results that the Doppler has limited impact on the algorithm when the relative speed between the transmitter and receiver is less than 0.5 m/s (assuming that the underwater acoustic velocity is 1500 m/s). We can solve the problem of the bit error rate by coding, such as Low Density Parity Check (LDPC) code.

### 4.2. Software Workflow

**Figure 13:**
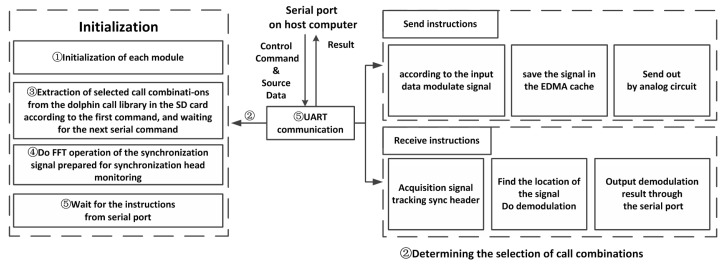
Software flowchart.

The software flowchart of this modem is shown in Figure 13. It mainly includes a DSP driver and algorithm realization.

The initialization of the system will be carried out after powering up the modem, including system PSC, interrupt, EDMA, IIC, AIC3106, serial port initialization, etc. In addition to the initialization of these modules, a few more steps are needed to complete the algorithm implementation.

To realize the algorithm on DSP, we take the whale clicks converted to sixteen bits of vector data. The data is stored in the SD card in the modem to realize the modulation and demodulation process in advance. In the initialization process, there are two serial communication processes between the Modem and host computer; the first one is to determine the selection of dolphin calls from the SD card and the second command is to determine the modem working in sending or receiving mode.

If it is in receiving mode, we will carry out the FFT process on the frame synchronization signal—it is mainly used in the fast correlation operation to find the synchronization signal and reduce the computing time. After completing all of the above steps, the system is initialized.

After the initialization, the whole system is working in a standby state, awaiting the transmission of serial instructions. If the system receives the sending instructions, it will continue to receive the data from the serial port and begin to carry out the algorithm modulation after receiving the cut-off character instruction.

If the system receives the instructions of data acquisition from the serial port, it begins to collect the signal and carries on the fast correlation operation. After finding the signal by seeking the correlation peak, the system demodulates the signal and the results are sent through the serial port to the host computer.

## 5. Experiment Result

**Figure 14:**
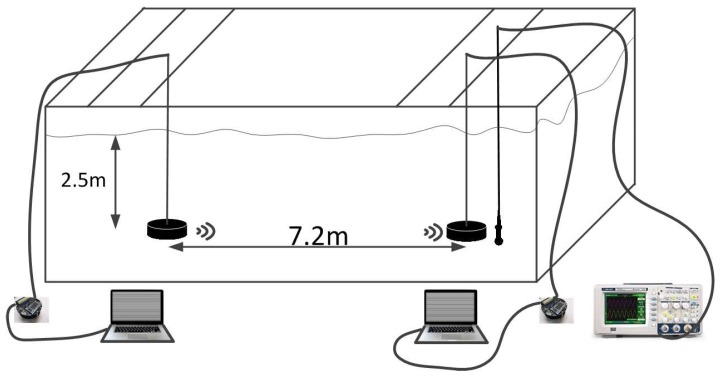
Schematic of the test environment.

To verify the functionality and performance of the PBC micro-modem, indoor experiments were conducted in a channel pool as shown in Figure 14. The pool length, width and depth are 45 m, 6 m, and 5 m respectively. For convenience, the transducers are only submerged in the water and the rest of micro-modem is placed on the desk in front of the water pool. The transducer and electronic part of the modem are connected by a cable. Also, each modem is connected to a corresponding computer via a RS-232 interface for control and monitoring.

According to the frequency of the click signal, we use the 2–8 kHz transducer and BRUEL &KJAER’s 8105 hydrophone to receive the signal. As the demodulation is mainly carried out by signal correlation, in order to reduce the bit error rate, we take the channel and noise effects into account. We removed the signal from the call library if the correlation coefficient between them is more than 0.30, retaining only the number c1, c2, c3, c4, c6 signals.

**Figure 15:**
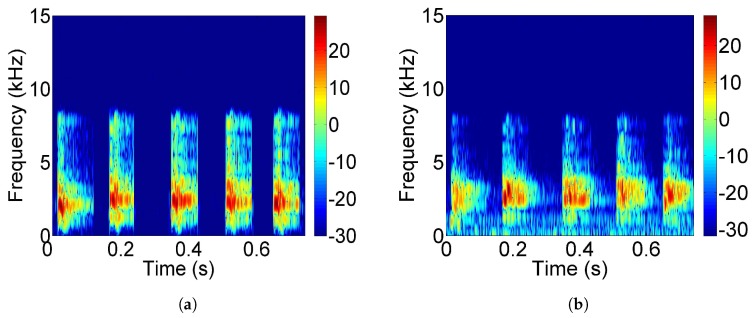
Comparison of the spectrogram of the call sample and the received signals; (**a**) The spectrogram of the dolphin calls sample; (**b**) The spectrogram of the received signals.

To evaluate the similarity between the dolphin-call samples and the received signals through the pool channel, we collected the received signals from the receiver, and the signal and call samples were compared in the spectrogram as follows. A comparison of the spectrogram of the call samples and the received signals is shown in Figure 15. From the spectrum of the received signal, it can be seen that there is multipath superposition on the signal, and there is some noise interference. After the signal passed through the pool channel, its spectrum changed; however, a certain extent of the spectrogram characteristics of dolphin calls are still maintained.

**Figure 16:**
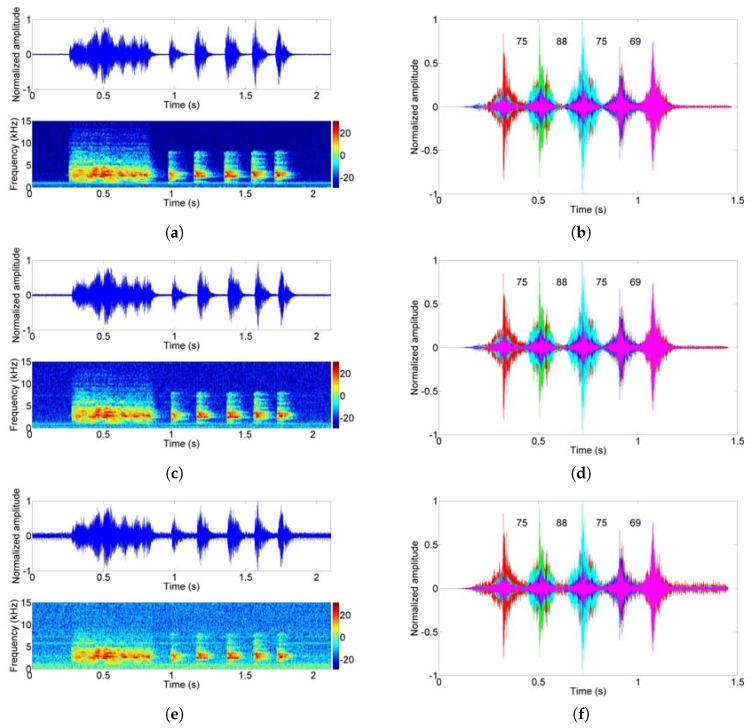
The time–frequency analysis and demodulation results; (**a**) Transmitted dolphin clicks; (**b**) Correlation result of transmitted clicks; (**c**) Received clicks at 7.2 m; (**d**) Correlation result of received clicks at 7.2 m; (**e**) Received clicks at 10 m; (**f**) Correlation result of received clicks at 10 m.

The time–frequency analysis and demodulation results are shown in Figure 16. Among them, the two signals were received at different distances (7.2 m and 10 m) and we reduced the transmit power by half at 10 m to further test the modem performance; the same information is carried for the purpose of contrast. During the two hours of testing, no error occurred and a data rate of 27.1 bits per second was achieved in the indoor trial.

The purpose of this experiment is to verify the algorithm and the performance of the modem. According to the specific needs, we can increase the distance between the clicks and the number of click signals.

## 6. Conclusions

In this paper, we have designed a portable micro-modem based on the bionic covert UAC algorithm for underwater sensor networks. Specifically, it is the first time that a modem has been used to communicate using real dolphin-call signals. It opens the door for secure and covert communication—the message between the divers or UUVs and control center cannot be identified. Additionally, the compact design of the modem has many advantages such as being portable for divers, it can be placed on UUV and it can also be used as a standalone unit for a particular application. In particular, it is suitable for combat divers’ information transmission at short distances and other military applications with covert operations. In the pool experiment, we tested the covert UAC algorithm by using the time delay between dolphin sounds and verified that the modem could efficiently perform reliable data transmission over short distances. At a distance of 10 m, a data rate of 27.1 bits per second is achieved. However, work is still in progress and our current work only uses transducers for lab communication. We plan to test our modem in the sea or a lake shortly. We will try to increase the communication distance as well as the data rate. We will also enhance the circuit of the modem, improve its stability, and reduce its output noise. The existing dolphin sounds library can be expanded, and more algorithms need to be developed to imitate dolphin sounds and realize covert communication. 

## Figures and Tables

**Table 1:** Comparison of developed devices, commercial devices and the Portable Biomimetics-based Covert (PBC) modem.

Underwater Acousitc Modem	Modulation	Areas of Application	Characteristics
Teledyne Benthos Atm 9xx [29]	PSK\MFSK	Remote Monitoring of Oceanographic Sensors Command of AUVs	Directional or Omni-directional High Data Rate
LinkQuest UWM series [30]	-	Short to Long Distance Communication Near-vertical or horizontal environments	Low Power Consumption Directional or Omni-directional High Data Rate
EvoLogics S2C M HS [31]	-	Short-range Shallow Waters Communication and Positioning for AUVs Diver tracking systems	Ultra-high-speed Smaller and Lighter
EvoLogics S2C R 7/17W [31]	-	Long-range Applications Underwater Observatories	Low Power Consumption Hemispherical Beam Pattern Long-term Deployment
WHOI micro-modem [5]	FH-FSK\PSK	UAC Navigation Subsystem	Variable Rate PSK Low Rate FH-FSK Long Base Line Navigation Narrow-band and Broadband
[23]	ASK	UWSN	Low Power Consumption Ultrasonic Wave Energy-aware Low Cost
[24]	ASK	UWSN	Omni-directional Beam Patter Low Power Consumption Low Cost Small Size
[25]	FSK	Long-life Short/Medium Range UWSN	Ultra-low Power Consumption Energy-efficient Low Cost
[26]	FSK	UWSN	High Efficient Low Cost Small Size
Our PBC modem	Unconventional mode	Short-range Covert Application Between Divers or UUVs	Covert Acoustic Communication by using Real or Mimetic- Cetacean Sounds Small size

**Table 2:** The normalized cross correlation and autocorrelation coefficients of each signal.

Signal	c1	c2	c3	c4	c5	c6	c7	c8	c9	c10
c1	1	0.13	0.15	0.11	0.17	0.13	0.38	0.16	0.24	0.16
c2	0.23	1	0.22	0.16	0.27	0.16	0.29	0.23	0.30	0.41
c3	0.22	0.19	1	0.23	0.28	0.14	0.24	0.31	0.37	0.20
c4	0.17	0.15	0.25	1	0.37	0.07	0.17	0.42	0.25	0.15
c5	0.14	0.13	0.16	0.19	1	0.07	0.13	0.15	0.20	0.18
c6	0.20	0.15	0.15	0.07	0.14	1	0.17	0.22	0.16	0.09
c7	0.43	0.19	0.18	0.12	0.17	0.11	1	0.37	0.14	0.15
c8	0.20	0.15	0.24	0.31	0.22	0.16	0.39	1	0.20	0.15
c9	0.20	0.15	0.22	0.14	0.21	0.09	0.11	0.15	1	0.27
c10	0.18	0.28	0.16	0.11	0.25	0.07	0.16	0.15	0.36	1
